# The role of the whitefly, *Bemisia tabaci* (Gennadius), and farmer practices in the spread of cassava brown streak ipomoviruses

**DOI:** 10.1111/jph.12609

**Published:** 2017-08-22

**Authors:** Midatharahally N. Maruthi, Simon C. Jeremiah, Ibrahim U. Mohammed, James P. Legg

**Affiliations:** ^1^ Natural Resources Institute University of Greenwich Chatham Maritime Kent UK; ^2^ Ministry of Agriculture, Livestock and Fisheries Development Ukiriguru Research Mwanza Tanzania; ^3^ International Institute of Tropical Agriculture (IITA) Dar es Salaam Tanzania; ^4^ Present address: Kebbi State University of Science and Technology Aliero (KSUSTA) Nigeria

**Keywords:** *Bemisia tabaci*, cassava, cassava brown streak disease, disease spread, virus transmission, whitefly

## Abstract

Cassava brown streak disease (CBSD) is arguably the most dangerous current threat to cassava, which is Africa's most important food security crop. CBSD is caused by two RNA viruses: *Cassava brown streak virus* (CBSV) and *Ugandan cassava brown streak virus* (UCBSV). The roles of the whitefly *Bemisia tabaci* (Gennadius) and farmer practices in the spread of CBSD were investigated in a set of field and laboratory experiments. The virus was acquired and transmitted by *B. tabaci* within a short time (5–10 min each for virus acquisition and inoculation), and was retained for up to 48 hr. Highest virus transmission (60%) was achieved using 20–25 suspected viruliferous whiteflies per plant that were given acquisition and inoculation periods of 24 and 48 hr, respectively. Experiments mimicking the agronomic practices of cassava leaf picking or the use of contaminated tools for making cassava stem cuttings did not show the transmission of CBSV or UCBSV. Screenhouse and field experiments in Tanzania showed that the spread of CBSD next to spreader rows was high, and that the rate of spread decreased with increasing distance from the source of inoculum. The disease spread in the field up to a maximum of 17 m in a cropping season. These results collectively confirm that CBSV and UCBSV are transmitted by *B. tabaci* semipersistently, but for only short distances in the field. This implies that spread over longer distances is due to movements of infected stem cuttings used for planting material. These findings have important implications for developing appropriate management strategies for CBSD.

## 1 INTRODUCTION

Cassava (*Manihot esculenta* Crantz) is a woody shrub that produces tuberous roots which are consumed as a staple in much of sub‐Saharan Africa (SSA). As well as being the main source of dietary calories for a large proportion of the rural and urban populations in SSA, cassava roots have an industrial use in the production of animal feed, starch, paper and biofuel (Nassar & Ortiz, 2007). The food security and livelihood benefits of cassava are, however, negatively affected by biotic constraints, of which the two most important are the virus diseases—cassava mosaic disease (CMD) and cassava brown streak disease (CBSD). CBSD currently has major impacts on production in eastern and southern African countries (Hillocks & Jennings, 2003; Legg et al., 2011, 2015). Until recently, CBSD was endemic only in the low altitude areas of Kenya, Malawi, Mozambique and Tanzania (Hillocks & Jennings, 2003; Storey, 1936, 1939) where the disease was reported to cause reductions of up to 70% in tuberous root yield of susceptible cultivars (Hillocks, Raya, Mtunda, & Kiozia, 2001). In addition to having direct deleterious effects on the growth of cassava plants, the disease causes necrosis of affected roots, making them unfit for consumption or marketing, and thus affecting food security (Legg et al., 2014). The continental significance of CBSD increased greatly from 2004, when the first reports were made of epidemics in mid‐altitude areas of Uganda (Alicai et al., 2007). In subsequent years, further outbreaks were reported from other countries in the Great Lakes region of East and Central Africa, including western Kenya, north‐western Tanzania, Rwanda, Burundi and Democratic Republic of Congo (Bigirimana, Barumbanze, Ndayihanzamaso, Shirima, & Legg, 2011; Legg et al., 2011; Mahungu, Bidiaka, Tata, Lukombo, & N'luta, 2003; Mulimbi et al., 2012). The disease has potential to spread from the mid‐altitude regions of East and Central Africa to the neighbouring cassava‐growing areas in southern and West Africa, and eventually to much of SSA with devastating consequences (Legg et al., 2014, 2015).

Cassava brown streak disease is caused by two distinct species of single‐stranded RNA (ssRNA) viruses: *Cassava brown streak virus* (CBSV) and *Ugandan cassava brown streak virus* (UCBSV), (genus *Ipomovirus*, family *Potyviridae*) (Mbanzibwa, Tian, Mukasa, & Volkonen, 2009; Mbanzibwa et al., 2011; Monger et al., 2010; Winter et al., 2010), which are together referred to as cassava brown streak ipomoviruses (CBSIs). Earlier work on the transmission of CBSIs showed that they can be graft‐transmitted from cassava to cassava (Ogbe, Dixon, Huges, Alabi, & Okechukwu, 2006) and mechanically transmitted from cassava to a number of herbaceous hosts (Lister, 1959; Mohammed, Abarshi, Muli, Hillocks, & Maruthi, 2012). In addition, it was suggested that CBSIs spread naturally in the field through the transmission activity of insects, in particular two whitefly species; *Bemisia tabaci* (Gennadius) (Bock, 1994; Storey, 1939) and *Bemisia afer* (Priesner & Hosny) (Hemiptera: Aleyrodidae), which were abundant in the CBSD endemic areas (Bock, 1994; Munthali, 1992). Subsequent transmission studies with both species of whitefly and with some species of aphid, however, were unsuccessful (Bock, 1994; Lennon, Aiton, & Harrison, 1986).

The first evidence of CBSV transmission by an insect vector, the whitefly *B. tabaci*, was obtained in our earlier laboratory studies (Maruthi et al., 2005), which was later confirmed (Mware et al., 2009). However, virus transmission patterns were inconsistent in both of these studies, and the low rate of transmission observed could not explain the high rate of spread in the field. The lack of correlation between laboratory studies and field observations has led to speculation that CBSIs may also be spread by other means, such as through contact between diseased and healthy plants, through tools contaminated during the process of cassava harvesting, and/or in the process of harvesting cassava leaves (leaf picking) for use as a vegetable.

The aim of this study was therefore to determine whether CBSIs can be transmitted by contaminated tools or during the process of leaf picking, as well as to understand the transmission characteristics of CBSIs by the *B. tabaci*. The findings from these studies will provide guidance for the development and implementation of control strategies to address what is currently one of Africa's biggest crop production threats.

## 2 MATERIALS AND METHODS

### 2.1 Cassava varieties, virus isolates and whitefly colonies used in the study

Two CBSD‐susceptible cassava varieties (var.)—Albert and TMS 60444—were grown from stem cuttings and confirmed to be free from CBSIs by reverse transcription polymerase chain reaction (RT‐PCR; Abarshi et al., 2010, 2012; Otti et al., 2016). These were used as target plants for virus inoculations in the UK. Two virus isolates—UCBSV from Kabanyoro, Uganda and CBSV from Naliendele, Tanzania—described previously were used in virus transmission experiments where indicated (Mohammed et al., 2012). Virus‐free plants of two cassava vars.—Kiroba and Kaleso—were also used to test the efficiency of virus transmission by whiteflies. Both Kiroba and Kaleso inhibit the multiplication of CBSV upon inoculation and were described as tolerant and resistant to CBSD, respectively (Maruthi, Bouvaine, Tufan, Mohammed, & Hillocks, 2014). Another cassava var. Ebwanateraka infected with either CBSV or UCBSV provided the source of viruses. The colony of *B. tabaci* used in this study was collected on cassava originally from Uganda and maintained subsequently on cassava in the quarantine insectary facilities of NRI in the UK (Maruthi, Colvin, & Seal, 2001). This colony was confirmed to belong to the species sub‐Saharan Africa 1‐subgroup 1 (SSA1‐SG1) based on mitochondrial cytochrome oxidase I gene sequences.

Virus‐indexed tissue culture plantlets of var. Kiroba, shown to be free of CBSIs using RT‐PCR, were hardened off in a screenhouse with insect‐proof netting in Kibaha, Pwani Region, Tanzania. These plants were subsequently used to establish the CBSD spread trials in the field and screenhouse in the year 2012, as described below. Field‐grown CBSD‐affected plants of the same cassava variety were obtained from field experiments at Kibaha for use as the spreader blocks in each of these trials, and *B. tabaci* adults used in this experiment were similarly obtained from field‐grown cassava plants.

### 2.2 Transmission of CBSV by *B. tabaci*

Initial CBSV transmission experiments by *B. tabaci* involved a combination of using long periods of virus acquisition access (AAP) and inoculation access (IAP) of up to 5 days and using high whitefly numbers to increase the probability of virus transmission. Whiteflies were collected from the colony and allowed to feed for 4 days on CBSD‐affected cassava plants of var. Ebwanateraka. The suspected viruliferous whiteflies were then collected and immediately released in two groups of either 20–25 or 50–100 on each healthy target plant for 5 days to inoculate the virus. In another experiment, between 25 and 100 whiteflies born on diseased plants were used for transmitting CBSV to each healthy plant (Table 1). Between 10 and 26 plants were inoculated for each category of whiteflies in three replications. All inoculated plants were enclosed individually in insect‐proof bread bags to prevent cross‐contamination. Plants were kept in an insectary (28 ± 5°C) and observed for symptom development. Unless otherwise specified, all plants used in controlled experiments in the UK were tested for infection with CBSV and UCBSV by RT‐PCR (Abarshi et al., 2010, 2012) three months after exposure to adult whiteflies from CBSD‐infected plants. Data on the number of plants infected with the viruses were subjected to Chi‐squared test using the software package sigmaplot for Windows version 11.0 (Systat Software inc., San Jose, CA, USA).

**Table 1:** Initial *Cassava brown streak virus* transmission experiments using the whitefly, *Bemisia tabaci*

No. of whiteflies used to inoculate each plant	AAP	IAP	No. of plants infected/inoculated	% transmission achieved
20–25	4 days	5 days	7/20	30.0
50–100	4 days	5 days	14/26	53.0
50–60	Whiteflies emerging from CBSD‐affected cassava plants	5 days	4/10	40.0

AAP, acquisition access period, IAP, inoculation access period.

### 2.3 Determining the mode of transmission of CBSV by *B. tabaci*

Transmission experiments were initiated to investigate potential non‐persistent, semipersistent and persistent modes of CBSV transmission by whiteflies. To verify the non‐persistent mode of transmission, whiteflies were given three relatively short AAP of 5–10 min, 30 min and 1 hr on a CBSV‐infected cassava plant of var. Ebwanateraka. About 20–25 adult viruliferous whiteflies were immediately introduced to each target plant for a 48 hr IAP.

To investigate the semipersistent mode of transmission, whiteflies were given a longer AAP of 24 and 48 hr on diseased plants, after which the suspected viruliferous insects were immediately transferred to healthy plants for a 48 hr IAP. Finally, to verify the persistent mode of transmission, whiteflies that had been introduced to healthy plants in the semipersistent experiment were collected and immediately transferred onto a new batch of healthy plants for 48 hr. Experiments were conducted in three replications, and between 15 and 25 plants were inoculated for each treatment (Table 2).

**Table 2:** Investigating the mode of *Cassava brown streak virus* transmission by the cassava whitefly, *Bemisia tabaci*

Mode of transmission tested	No. of whiteflies per plant	AAP	IAP	No. of plants infected/inoculated	% transmission achieved
Non‐persistent mode of transmission	20–25	5–10 min	48 hr	3/25	12.0
	20–25	30 min	48 hr	5/25	20.0
	20–25	1 hr	48 hr	4/25	16.0
Semipersistent mode of transmission	20–25	24 hr	48 hr	5/20	25.0
	20–25	48 hr	48 hr	8/20	40.0
Persistent mode of transmission^a^	10–20	24 hr	48 hr + 48 h	0/15	0
	7–20	48 hr	48 hr + 48 hr	0/15	0

AAP, acquisition access period; IAP, inoculation access period.

a This was investigated by allowing the suspected viruliferous whiteflies to feed on a batch of healthy cassava plants for 48 hr. The whiteflies were then transferred to a new batch of cassava plants to investigate the persistence of CBSV in adult *B. tabaci*.

Chi‐squared analyses of data from these experiments were conducted in all possible combinations to identify significant differences between the treatments (Table 2). All non‐persistent treatments were compared to all persistent and semipersistent treatments (both 24/48 and 48/48 hr AAP/IAP combinations). Finally, all semipersistent treatments were compared to all persistent treatments using sigmaplot 11.0.

### 2.4 Determining virus acquisition, inoculation and retention times in *B. tabaci*

For testing AAP, whiteflies were allowed to feed on CBSV‐infected cassava var. Ebwanateraka for 5–10 min, 30 min, 1 hr, 4 hr, 24 hr and 48 hr. Other whiteflies tested had emerged from the nymphal stage on infected plants. For each category of AAP, 20–25 suspected viruliferous whiteflies were immediately transferred to between 15 and 25 healthy plants of var. Albert for 48 hr IAP in three replications.

The methodology used to estimate IAP was similar to that of AAP except that the time given for whiteflies to inoculate the virus varied and included the following time periods: 5–10 min, 30 min, 1 hr, 4 hr, 24 hr, 48 hr and up to death (which was on average 15 days). Each category of whiteflies was given a 48 hr AAP on diseased cassava plants prior to inoculation. Between 31 and 48 plants were inoculated in four replications for each category of 24 hr IAP or less (Table 3). A total of 15 plants were inoculated for the category 48 hr IAP with three replications.

**Table 3:** Determining AAP and IAP of *Cassava brown streak virus* in the cassava whitefly, *Bemisia tabaci* a

Time period	Determining AAP for CBSV on cassava^b^		Determining IAP for CBSV on cassava^c^	
	Total no. of plants infected/inoculated	% infected plants	Total no. of plants infected/inoculated	% infected plants
5–10 min	4/25	16.0	6/31	19.3
30 min	8/25	32.0	7/33	21.2
1 hr	10/25	40.0	8/39	20.5
4 hr	6/15	40.0	13/35	37.1
24 hr	9/20	45.0	29/48	60.4
48 hr	6/15	40.0	6/15	40.0

a About 20–25 viruliferous whiteflies inoculated each plant in this experiment.

b Suspected viruliferous whiteflies were given a standard 48 hr inoculation access period (IAP) for testing different acquisition access periods (AAPs).

c Suspected viruliferous whiteflies were given a standard 48 hr acquisition access period (AAP) for testing different inoculation access periods (IAPs).

To determine the retention of CBSV by whiteflies, insects were given a 24 hr AAP on diseased cassava plants after which they were immediately transferred to healthy cassava plants for an IAP of 24 hr or 48 hr. The surviving insects from the 24 hr or 48 hr IAP plants were then collected and re‐released on to a new batch of healthy cassava plants for a further 48 hr to verify if the whiteflies retained CBSV following feeding on healthy plants.

Similar to the previous experiments, several combinations of treatments (Table 3) were compared using Chi‐squared tests to identify the effect of various time points on virus transmission efficiencies. For the AAP, 5–10 min vs. 1 hr plus, 30 min vs. 1 hr plus and 5–10 min vs. 30 min values were compared. For IAP, ≤1 hr vs. ≥4 hr and 4 hr vs. 24 hr values were compared.

### 2.5 Determining other virus‐vector transmission parameters

Cassava brown streak disease produces typical chlorotic symptoms on older leaves at the bottom of infected plants, while the younger leaves are either symptom‐free or only show early symptoms of the disease (vein clearing but not yellowing). How this affects virus acquisition and subsequent transmission by the whiteflies was not known. To investigate this, groups of whiteflies were confined in small plastic cages for a 48 hr AAP on mature symptomatic leaves at the bottom of the plant, or on younger leaves showing early signs of CBSD symptoms. Suspected viruliferous whiteflies were collected and then immediately allowed to feed freely on healthy plants of var. Albert for 48 hr for virus inoculation to determine the effect of leaf age on virus transmission. A total of seven and 22 plants were inoculated for the older and younger leaf categories, respectively. The transmission efficiencies of CBSV and UCBSV were also compared using 20–25 whiteflies per plant that were given a 48 hr AAP and IAP each. A total of 15 and 29 plants were inoculated for CBSV and UCBSV, respectively, in three replications.

### 2.6 Transmission of CBSV and UCBSV to different cassava varieties

Three cassava var—Albert, Kiroba and Kaleso—were inoculated with CBSV or UCBSV by whiteflies to validate the whitefly transmission method for varieties with contrasting levels of resistance to CBSD. Albert is susceptible to CBSD, Kiroba is tolerant with delayed expression of root symptoms, and Kaleso is resistant with no root symptoms but with mild leaf symptoms. Negligible amounts of virus accumulate in Kaleso and Kiroba, while high amounts of virus accumulate in Albert (Maruthi et al., 2014). Thirty plants of each variety were each inoculated with 20–25 suspected viruliferous whiteflies that were given an AAP and IAP of 24 hr each. The experiment was conducted in three replicates for each virus‐variety combinations.

### 2.7 Mechanical transmission of CBSV and UCBSV

Three methods of transmission were investigated for CBSV and UCBSV in a set of experiments by sap inoculation, transmission by leaf picking and contaminated tools. Cassava plants of var. Albert and TMS60444 were each inoculated with sap extracted from either CBSV‐ or UCBSV‐infected cassava plants in 0.06 m potassium phosphate buffer (Mohammed et al., 2012). To minimize the effects of experimental variables on the sap transmission of the viruses, the top two fully expanded leaves of the test plants of uniform age group (2 months old) were used for both virus species. Source of the virus inoculum was obtained from a single cassava var. Ebwanateraka for both virus species. A total of 120 plants were inoculated in this experiment, which contained three replications with 10 plants in each replication for each virus species (3 replications × 10 plants × 2 varieties × 2 virus species = 120). Plants inoculated with buffer alone served as controls. The efficiency of sap transmission of UCBSV and CBSV was determined by assessing the presence or absence of the virus by RT‐PCR. Unless otherwise stated, one leaf from leaf numbers 3–5 from the top of the plants was used as a sample for testing for virus infections by RT‐PCR (Abarshi et al., 2010, 2012; Otti et al., 2016).

Shoots of cassava plants containing tender leaves are picked/snapped in some countries of SSA for use as a leafy vegetable. We mimicked this process by picking leaves alternately between virus‐infected and virus‐free plants of three‐month‐old var. Albert and TMS60444. This was done in an attempt to transmit the virus from diseased to healthy plants by hands that become contaminated with plant sap in the process of leaf picking. Similar to the above experiments, a total of 120 plants were used in the experiment and tested for virus infection by RT‐PCR after 6 months. Leaf picking between healthy plants served as a control.

Farmers use machetes for cutting stems of cassava plants to produce stem cuttings for planting material. We imitated this process by alternately cutting stems of virus‐infected and virus‐free cassava plants of var. Albert and TMS60444 using a pair of secateurs. A single cut to the stem of an infected stem was followed by a cut to the stem of a healthy plant of the same variety. Following this process, 30 cuttings were made for each variety and virus type in a three replicate experiment, giving a total of 120 inoculated plants. Ten plants of each variety cut between virus‐free plants only used as a control. The 3rd to 5th leaf from the top of the plant was used for testing for virus infection by RT‐PCR after 6 months (Abarshi et al., 2010, 2012). Data from the above three experiments were compared using the ANOVA procedure in sigmaplot 11.0.

### 2.8 Screenhouse simulation of CBSD spread

A 20 m × 8 m insect‐proof screenhouse, at Kibaha Research Station, Kibaha, Tanzania, was used in the year 2012 to establish an experiment that aimed to simulate field‐based spread of CBSD. In one half of the screenhouse, a spreader plot of CBSD‐infected cuttings (var. Kiroba) was planted in the soil using a spacing of 0.5 m × 0.5 m. Once these plants had sprouted, virus‐free cuttings obtained from virus‐indexed tissue culture plants of var. Kiroba were planted in 10 L pots in the second half of the screenhouse. These were arranged in four blocks of 60 plants each, at increasing distances from the spreader, with block 1 closest to the spreader, and block 4 furthest away. Each block was further divided into four replicates, each of which comprised three rows of five plants. Plants within replicates were spaced at 0.5 m × 0.5 m, while there were 1 m gaps between replicates and between blocks. The central rows of each block were 2 m (block 1), 4 m (block 2), 6 m (block 3) and 8 m (block 4) distant from the closest row in the spreader plot.

Four weeks after the potted test plants had been planted (4 WAP), >1,000 field‐collected adult *B. tabaci* were introduced to the central part of the spreader plot. Whiteflies were subsequently able to move freely from plant to plant and through the screenhouse. From 4 WAP, and at approximately weekly intervals, CBSD symptom presence/absence, CBSD severity and whitefly abundance were recorded for all test plants as described previously, and for the spreader plot row closest to the test plants.

*Bemisia tabaci* population increase on the spreader plot began to produce physical damage to spreader plants from 13 WAP, so these plants were cut back to 15 cm above ground level (ratooned) and allowed to resprout. This action had the additional intended effect of encouraging movement of whiteflies from the spreader to the test plots. Record taking resumed approximately 1 month after ratooning, and was continued for an additional 5 weeks. The ANOVA procedure of sigmaplot 11.0 was used to analyse the pattern of distribution between plots of both CBSD incidence and whitefly abundance, while Pearson's correlation and linear regression analyses were employed to examine the relationship between whitefly abundance and CBSD.

### 2.9 Field transmission of CBSIs

A field experiment was established in 2012 at Kibaha Research Station, Kibaha, Coast Region, Tanzania, to examine the spatiotemporal pattern of CBSD spread into initially CBSD‐free plants. Tissue culture derived plants of var. Kiroba were hardened off in an insect‐proof screenhouse before being planted out in the field as stem cuttings in an experimental trial. The experiment was planted in an isolated location, surrounded by natural vegetation (uncultivated) and more than 300 m away from the nearest field of cassava. The trial comprised one 50‐plant “spreader” plot and five test plots each containing 20 plants. All plots were planted at the standard spacing of 1 m × 1 m. The spreader plot was planted with 10 rows of five plants each, and cuttings used for this plot were obtained from CBSD‐affected parent plants. Each of the five test plots was made up of four rows of five plants, and there was a spacing of 2 m between all plots. One test plot was adjacent to the spreader. Other test plots were situated on the distal side of the first test plot with respect to the spreader, and at increasing distances from it (2 m from spreader, 7 m, 12 m, 17 m and 22 m).

The spreader plot was planted 1 month before the test plots to encourage vector spread from the spreader to the neighbouring test plots. Although whiteflies were able to enter the experiment from the surrounding area, planting the spreader before the test plots ensured that there was movement of whiteflies from the spreader to the test plots, as whiteflies typically move from older to younger plants. Starting at 2 months after test plot planting (2 MAP), records were taken for all test plot plants of the presence/absence of foliar CBSD symptoms, the severity of those symptoms and numbers of the whitefly vector, *B. tabaci*. Severity was assessed using the standard 1–5 scoring system in which “1” corresponds to symptom‐free, “2” to the mildest symptoms and “5” the most severe symptoms (Hillocks & Jennings, 2003; Hillocks et al., 2001). Whitefly abundance was assessed by counting the number of adult *B. tabaci* on the top five leaves of each plant. Unless otherwise indicated, data were recorded at weekly intervals up to 6 MAP. Kruskal–Wallis one‐way analysis of variance on ranks was used to test for the significance of gradients in CBSD incidence and whitefly abundance from the nearest to the farthest plot from the spreader. For this test, data for each of the time points (from 4 WAP to 22 WAP) were considered as replicates.

## 3 RESULTS

### 3.1 Verifying the transmission of CBSV by *B. tabaci*

Highest virus transmission was recorded (53.0%) when 50–100 whiteflies that had up to 5 days each AAP and IAPs were used in the experiments (Table 1). Rate of transmission was less (40.0%) when 50–60 whiteflies that emerged from CBSD‐affected cassava plants inoculated each target plant. The efficiency of transmission was further reduced (to 30.0%) when only 20–25 whiteflies were used. No significant differences were observed in CBSV transmission efficiencies for any of the three initial tests which had different numbers of whiteflies (χ^2^ = 1.731; *p* = .421, *df* = 2). Although the highest rate of transmission was achieved using a large number of insects (50–100), we used 20–25 whiteflies in subsequent experiments to prevent feeding damage to the test plants caused by high whitefly numbers.

### 3.2 Mode of CBSV transmission by *B. tabaci*

Whiteflies that had an AAP of 5–10 min were able to acquire and transmit CBSV to 12.0% of inoculated plants. Whiteflies that had 30 min and 1 hr AAP transmitted CBSV to 20.0% and 16.0% of the plants, respectively (Table 2). The rate of transmission increased to 25.0% and 40.0% with the increase in AAP to 24 and 48 hr, respectively. Suspected viruliferous whiteflies that were previously fed on healthy cassava plants for 24 or 48 hr did not transmit CBSV to the second batch of healthy cassava plants, indicating that whiteflies lost the virus within 24 hr after virus acquisition (Table 2).

No significant differences were observed in CBSV transmission efficiencies when comparing all non‐persistent treatments with 24/48 AAP/IAP semi‐persistent treatments (χ^2^ = 0.37; *p* = .55, *df* = 1). Significant differences were seen between non‐persistent treatments and 48/48 AAP/IAP semi‐persistent treatments (χ^2^ = 4.12, *p *=* *.042, *df* = 1), and between all non‐persistent and all persistent treatments (χ^2^ = 3.95, *p *=* *.047, *df* = 1). Strongly significant differences were observed when comparing all semipersistent treatments to all persistent treatments (χ^2^ = 9.92, *p* = .002, *df* = 1).

### 3.3 AAP, IAP and retention of CBSV in *B. tabaci*

This experiment reconfirmed that CBSV can be acquired within 5–10 min of whitefly feeding on CBSD‐affected plants (Table 3). Highest rate of transmission (45.0%) was achieved at 24 hr AAP, although this was not significantly different from those that had AAPs of 1 hr, 4 hr and 48 hr. Whiteflies were also able to transmit CBSV within 5–10 min (IAP) of feeding on a healthy plant (Table 3). The highest rate of transmission (60.4%) was achieved when feeding for 24 hr. In the experiment to determine the retention of CBSV by the vector, whiteflies were given a 24 hr AAP on CBSD‐affected cassava plants. None of the suspected viruliferous whiteflies fed on healthy cassava plants for 48 hr and subsequently transferred to a new batch of healthy cassava plants transmitted CBSV, again confirming that whiteflies had lost the ability to transmit the virus by 48 hr after acquisition.

Comparison of data by Chi‐squared tests showed significant differences in transmission efficiencies between whiteflies with 5–10 min AAP and those with 1 hr plus AAP (χ^2^ = 4.23, *p *=* *.04, *df* = 1). However, no significant differences were seen between 5–10 min and 30 min AAP (χ^2^ = 0.99, *p *=* *.32, *df* = 1), and 30 min and 1 hr plus AAP (χ^2^ = 0.35, *p *=* *.55, *df* = 1). Highly significant differences were obtained when comparing 1 hr or less IAP vs. 4 hr or more IAP (χ^2^ = 16.96, *p *< .001, *df* = 1), while the comparison between 4 hr IAP vs. 24 hr IAP was not significant (χ^2^ = 3.51, *p *=* *.061, *df* = 1).

### 3.4 Effect of leaf age, virus species and cassava variety on virus transmission

Whiteflies that fed on younger leaves with no or early symptoms of CBSD achieved a slightly higher rate of transmission (36.3%) compared to those fed on older but fully symptomatic leaves (28.5%). Between 5 and 12 plants were inoculated in each of the three replications in the experiment conducted to compare the transmission efficiencies of the two viruses. The rate of CBSV transmission achieved (40.0%, mean number of plants infected ± standard deviation 3.3 ± 1.15) was slightly greater than that of UCBSV (34.5%, 2.0 ± 1.00), although this difference was not statistically significant. The rate of transmission also varied when cassava varieties differing in disease resistance levels were challenged by whitefly inoculations. There were statistically significant differences in the transmission of CBSIs to the three cassava varieties tested (*F* = 29.7; *p *<* *.001). Kaleso was less affected than the other two varieties, but there was no significant difference between Albert and Kiroba.

The mean numbers of plants infected in three replications of 10 plants each, with standard deviation and transmission rate, respectively, for each virus‐variety combination were as follows: CBSV infecting: Albert (mean ± *SD* = 5.6 ± 0.58, transmission rate 56.6%), Kiroba (4.6 ± 1.53, 46.6%) and Kaleso (0.3 ± 0.58, 3.3%), and for UCBSV infecting: Albert (5.0 ± 2.00, 50%), Kiroba (4.3 ± 1.53, 43.3%) and Kaleso (0.0 ± 0.00, 0%).

### 3.5 Verifying non‐vector transmission of CBSIs

Cassava brown streak virus, but not UCBSV, was transmitted at low levels by sap inoculation from infected cassava to virus‐free cassava plants (χ^2^ = 11.20, *p *< .001, *df* = 1). Only 16.6% of Albert and 23.3% of TMS 60444 plants took CBSV infection. A period of up to 8 weeks was required for CBSD symptom expression on the sap‐inoculated plants.

In the experiment conducted to verify the transmission of CBSIs by leaf picking, none of the tested plants from var. Albert and TMS 60444 expressed CBSD symptoms for the two viruses. All plants were also negative for CBSIs when tested by RT‐PCR.

Similarly, none of the plants showed CBSD symptoms 6 months after planting in the experiment conducted to verify the transmission of CBSIs by contaminated secateurs. The viruses were also not detected by RT‐PCR in these plants.

### 3.6 Screenhouse simulation of CBSD spread

#### 3.6.1 Whitefly abundance

**Figure 1:**
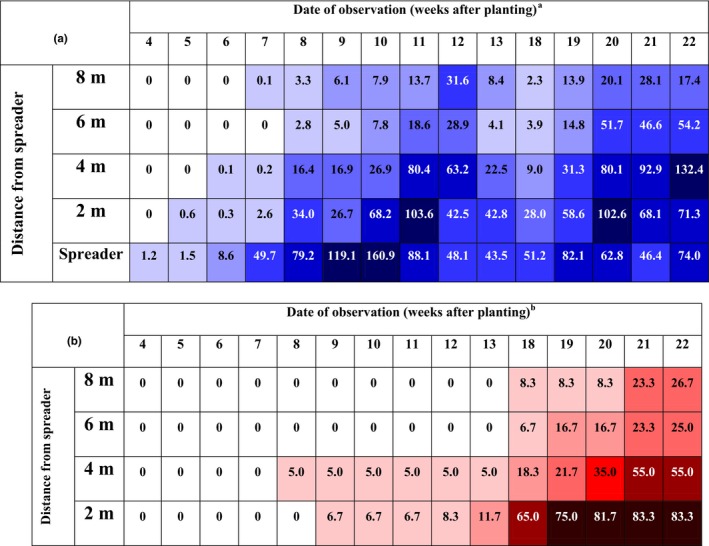
Spatiotemporal distribution of *Bemisia tabaci* (a) and cassava brown streak disease (b) on initially disease‐free cassava plants under screenhouse, Kibaha, Tanzania, shown as a heat map. ^a^Values in boxes are mean numbers of adult *B. tabaci* per plant. The figure is a heat map ‐ the increased intensity of the colour indicates increased number of *B. tabaci* adults per plant. ^b^Values in the boxes are percent CBSD incidence. The figure is a heat map ‐ the increased intensity of the colour indicates increased CBSD incidence [Colour figure can be viewed at wileyonlinelibrary.com]

Whiteflies were first recorded from test plots 1 week after their introduction, but over the course of the first 4 weeks of records (4–7 WAP) spread to reach block 4, which was most distant from the spreader (Figure 1a). This means that in the absence of wind in the protective environment of a screenhouse, whiteflies took 7 weeks to move from spreader rows to the farthest block. By 8 WAP, a strong abundance gradient was established running from block 1 to block 4, and this was maintained up to 11 WAP. Throughout this period, whiteflies were therefore most abundant in the block nearest to the spreader, and least abundant in the block farthest from the spreader. Whitefly abundance declined just before the spreader plot was ratooned (13 WAP), but then increased again from 18 WAP up to the final three weekly records (20–22 WAP). ANOVA results demonstrated a clear gradient in whitefly abundance at 18 WAP running from block 1 (highest) to block 4 (least) (Table 4; *F* = 10.0, *p *<* *.001, *df* = 15), but there were no significant differences between blocks by the time of the final data record at 22 WAP (*F* = 1.1, *p *=* *.38, *df* = 15). Whiteflies had therefore become evenly dispersed throughout the screenhouse by the end of the experiment.

**Table 4:** Incidence of cassava brown streak disease and *Bemisia tabaci* abundance in a screenhouse at Kibaha Research Station, Tanzania^a^

Distance from spreader (m)	CBSD incidence (*SE*) 18 WAP	CBSD incidence (*SE*) 22 WAP	Whitefly abundance (*SE*) 18 WAP	Whitefly abundance (*SE*) 22 WAP
2	65.0a (7.4)	83.3a (4.3)	28.0a (5.5)	71.3a (42.8)
4	18.3b (5.0)	55.0b (4.2)	9.0b (4.4)	132.4a (79.4)
6	6.7b (4.7)	23.3c (9.6)	3.9b (2.2)	54.2a (13.1)
8	8.3b (6.3)	26.7c (7.2)	2.3b (1.3)	17.4a (2.8)

a Means compared using the Holm–Sidak procedure. Values with different letters were significantly different at the *p *=* *.05 level. Values in brackets are standard errors (*SE*). WAP—weeks after planting. Incidence values are percentages.

#### 3.6.2 CBSD incidence

The first symptoms of CBSD in test plants were recorded in block 2 at 8 WAP (Figure 1b). CBSD was restricted to blocks 1 and 2 (maximum distance 4 m) up to 13 WAP. Incidences increased greatly in all blocks following the ratooning of the spreader—from 18 WAP onwards. There were strong gradients in the incidence of CBSD from the nearest (highest incidence) to the furthest (lowest incidence) blocks away from the spreader from 18 to 20 WAP, after which the disease became more generally distributed (Figure 1a). Statistically significant gradients were seen in CBSD incidences for both the 18 WAP and 22 WAP data sets (Table 4).

**Figure 2:**
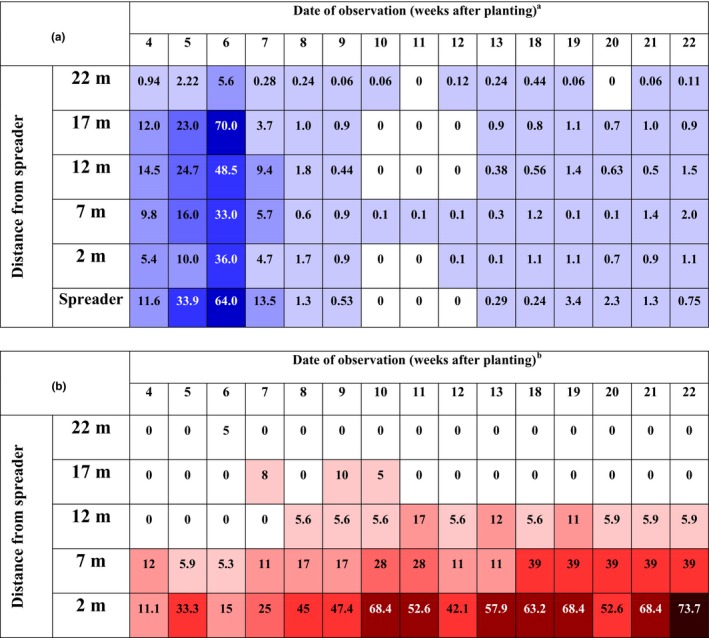
Spatiotemporal distribution of *Bemisia tabaci* (a) and cassava brown streak disease (b) on initially disease‐free cassava plants in the field, Kibaha, Tanzania, shown as a heat map. ^a^Values in boxes are mean numbers of adult *B. tabaci* per plant. The figure is a heat map ‐ the increased intensity of the colour indicates increased number of *B. tabaci* adults per plant. ^b^Values in the boxes are percent CBSD incidence. The figure is a heat map ‐ the increased intensity of the color indicates increased CBSD incidence [Colour figure can be viewed at wileyonlinelibrary.com]

It was evident both from the graphical representation of the data (Figures 1 and 2) and the statistical analyses (Table 4) that gradients in whitefly abundance corresponded with those for CBSD incidences. To examine this further, Pearson's correlation analyses were run to relate mean whitefly abundances to CBSD incidences for corresponding plots, using both the 18 WAP and 22 WAP data sets (Table 5). The strongest correlation was obtained with whiteflies at 18 WAP and CBSD at 22 WAP. In addition, there was a strongly significant linear regression relationship between whitefly abundance at 18 WAP and CBSD incidence 4 weeks later (CBSD = 0.28 + 0.018 WF; *F* = 24.0, *p *<* *.001, *r* ^2^ = .63).

**Table 5:** Pearson's correlation analyses relating *Bemisia tabaci* abundance with cassava brown streak disease incidence for the 16 test plots (four per block) within the screenhouse trial, Kibaha, Tanzania

Comparison	*R*	*p* ^a^	*N*
Wf 18 WAP vs. CBSD 18 WAP	0.77	.0006***	16
Wf 22 WAP vs. CBSD 22 WAP	0.29	.27ns	16
Wf 18 WAP vs. CBSD 22 WAP	0.80	.0002***	16

ns, not significant; Wf, whiteflies; CBSD, cassava brown streak disease; WAP, weeks after planting.

***p = highly significant, at .001 level.

### 3.7 Field spread of CBSD

#### 3.7.1 Whitefly abundance

The number of whiteflies ranged from 5 to 15 per plant through the entire plot when recording started at 4 WAP, with the exception of the most distant plot from the spreader in which whitefly abundance was generally low for the duration of the experiment (Figure 2a). The numbers increased steadily and reached a maximum of 70 adults per plant by 6 WAP. They then decreased gradually reaching almost zero in the period from 10 to 12 WAP. This population reduction corresponded with a prolonged cool and dry period occurring in the main dry season of coastal Tanzania. The whitefly numbers never subsequently recovered and on average numbered 1–2 insects per plant for the duration of the experiment (22 WAP). The Kruskal–Wallis ANOVA on ranks test comparing whitefly abundance in each of the experimental plots provided no evidence for differences between plots (*H* = 6.8, *df* = 4, *p *=* *.150). It was therefore concluded that whiteflies were randomly distributed between plots.

#### 3.7.2 CBSD incidence

The first symptoms of CBSD on test plants were recorded at 4 WAP, 2 m and 7 m from the spreader plot (Figure 2a). Incidences of CBSD appeared at 17 m from the spreader plot starting from 7 WAP. The first symptoms at 12 m from the spreader plot were observed at 8 WAP. There was a strong gradient of declining CBSD incidence from the test plot nearest to the spreader plot (2 m) to the plot that was 12 m from the spreader (Kruskal–Wallis ANOVA on ranks: *H* = 60.2, *df* = 4, *p *<* *.001). Tukey's test pairwise comparisons derived from the Kruskal–Wallis analysis showed that CBSD incidence in the 2 m plot was greater than those in the 12 m, 17 m and 22 m plots, while incidence in the 7 m plot was greater than those in the 17 m and 22 m plots. The CBSD incidence gradient was sustained from 7 WAP to the end of the experiment at 22 WAP. Disease incidences were generally low at 12 and 17 m from the spreader, and CBSD was not recorded at all in the 22 m plot.

## 4 DISCUSSION

Research into CBSD and its causal viruses (CBSV and UCBSV) has increased greatly as the spread of the disease was reported into previously unaffected parts of East Africa (Alicai et al., 2007). However, the mechanisms of transmission of these viruses remain poorly characterized. Our results respond to several of the key questions on transmission and epidemiology. Initial experiments confirmed that CBSV can be transmitted by *B. tabaci* adults under laboratory conditions. The rate of transmission, however, was moderate (highest 53%) even when using high whitefly numbers (50–100 per plant) and with prolonged acquisition and inoculation access periods of up to 5 days, or when using whiteflies that had emerged from CBSD‐affected plants. These results were, however, similar to previous findings (Maruthi et al., 2005; Mware et al., 2009) and further confirmed the generally moderate efficiency of CBSV transmission by *B. tabaci*. Experiments investigating the time required for virus acquisition revealed that CBSV can be acquired within 5–10 min of feeding on diseased plants, although the rate of transmission achieved from this short AAP was low (12%). Increasing the AAP to 24 hr resulted in significantly increased transmission efficiency (45%), although efficiency of transmission was similar for all AAPs between 1 and 48 hr. The shortest time period used (5–10 min) for IAPs resulted in 19% infected plants, confirming that CBSV can be both acquired and inoculated in very short periods of time. Notably, the combination of an AAP of 48 hr with an IAP of 24 hr resulted in 60% of plant infections, which represents a relatively high level of transmission efficiency. When suspected viruliferous whiteflies were placed on uninfected host plants for 24 or 48 hr, and then transferred to a further set of uninfected host plants for 48 hr, no infections result. This suggests that *B. tabaci* do not retain CBSV for long after leaving infected plants. Put together, our results indicated that CBSV is semipersistently transmitted by *B. tabaci*. The transmission of CBSV, by contrast, seems to be comparable to other whitefly‐transmitted ipomoviruses such as *Squash vein yellowing virus* (SqVYV) in the USA (Webb, Adkins, & Reitz, 2012) and *Cucumber vein yellowing virus* (CVYV) in the Middle‐East (Harpaz & Cohen, 1965; Mansour & Al‐Musa, 1993). SqVYV was acquired and transmitted in 30 min with moderate transmission efficiency (50%) using 25–35 whiteflies per plant at 24 hr AAP and 24 hr IAP. Whiteflies' retention of SqVYV declined rapidly after they were removed from infected plants (infection rate dropped from 76% to 20% after 1 hr), and they lost the ability to transmit the virus completely within 8–24 hr (Webb et al., 2012). Transmission of CVYV was also moderately efficient. Virus acquisition and inoculation occurred within 10–20 min, but required 30–35 whiteflies to reach a highest transmission rate of 80%. Persistence in the vector was also short, with a dramatic decrease in transmission from 81% to 14% after 2 hr (Harpaz & Cohen, 1965). Similar results were obtained using another isolate of CVYV in the 1990s (Mansour & Al‐Musa, 1993), indicating that regardless of the geographical location, the different whitefly species used in transmission experiments or the host plants they infect—ipomoviruses are generally transmitted with only moderate efficiency by their whitefly vectors and are only retained for short periods after the removal of the vector from an infected host.

Experiments comparing the transmission of two CBSD‐causing viruses—CBSV and UCBSV—showed that both were transmitted to the susceptible var. Albert as well as to the resistant vars. Kiroba and Kaleso, although at differing efficiencies. UCBSV was only transmissible to Albert and Kiroba, but not to Kaleso. This could be due to the relatively mild nature of the virus and low virus quantities in infected plants (Mohammed et al., 2012; Winter et al., 2010). CBSV in comparison was transmitted to all three varieties with different efficiencies, including the resistant var. Kaleso, confirming that whiteflies play a significant role in virus spread in the field irrespective of the variety that is grown. Experiments confirmed that neither leaf picking nor the use of contaminated tools for cutting stems resulted in CBSV transmission. It is therefore concluded that neither of these widespread practices contribute to the epidemiology of CBSD in the field, as had been suspected by some researchers. Circumstantial evidence further confirms this finding, as leaf picking is practiced in some regions of East Africa and not in others, and there is no apparent association between the incidence of CBSD and the prevalence of leaf picking. Similarly, if stem cutting resulted in transmission, significant increases in incidence might be anticipated even in areas where whiteflies are infrequent, which does not match with field data (Jeremiah et al., 2015; Legg et al., 2011).

Cassava brown streak virus was poorly transmitted by mechanical inoculation of sap extracted from diseased cassava leaves, while UCBSV was not transmitted at all, further indicating that this might be to do with the relatively low titres in infected plants or mild nature of the virus. Epidemiology experiments run in both confined screenhouse and open field conditions in coastal Tanzania showed that CBSD spread along a clearly defined gradient from CBSD‐affected spreader plots. The gradient of spread was relatively steeper in the screenhouse, probably as whiteflies were initially introduced from only one side (in the spreader plot) and wind speeds were low. The clear gradient in whitefly abundance demonstrated in the screenhouse was absent in the field experiment, almost certainly because they moved naturally from the surrounding vegetation into the field experiment. In both experiments, there was a clear association between the abundance of *B. tabaci* whiteflies and new CBSD infections, both in space and through time. Over the 8 months that data were recorded in the field experiment, the furthest distance that CBSD infections were recorded from the spreader plot was 17 m. Both experiments emphasize the relatively short distances over which CBSIs are spread—a result which is strongly congruent with the semipersistent transmission mechanism described from the laboratory experiments. Whiteflies migrated into the field experiment randomly from the surrounding vegetation. However, the strong gradient of CBSD between the spreader and the test plots, in which no CBSD at all was recorded from the test plot furthest away from the spreader, provides clear evidence that the spreader plot was the only significant source of CBSD. The corollary of this is that neither the natural vegetation immediately surrounding the field experiment, nor the distant (>300 m) cassava fields that had significant incidences of CBSD, had any significant effect on CBSD spread in the test plots of the field experiment.

The results of our experiments present a consistent picture for the pattern of transmission of CBSIs by the whitefly vector—*Bemisia tabaci*. As well as helping to explain how CBSD is spreading, knowledge of the semipersistent transmission mechanism also allows us to design appropriate and effective control strategies. The relatively poor retention of CBSIs by *B. tabaci*, and associated short gradients of spread, means that isolation is likely to be more effective in preventing infection from neighbouring virus sources. Using this as a basis, a novel cassava phytosanitation programme has been implemented in Tanzania to remove all CBSD‐affected cassava from rural communities to establish CBSD‐free zones. Farmers are then given disease‐free cassava planting material for cultivation, which is expected to remain disease‐free because of the poor transmission of CBSIs by the whiteflies. If implemented together with the development and dissemination of disease‐resistant cassava varieties, this can be a successful strategy for CBSD control in affected countries. Our results also indicate that by far the greatest threat of long‐distance spread of CBSIs comes from the inadvertent carriage by people of infected cassava stems. This will require implementing stricter quarantine regulations to prevent the movement of infected cassava material as well as applying rigorous phytosanitary standards when multiplying and disseminating cassava germplasm obtained from regions affected by CBSD.
